# Enamel Surface Remineralization Effect by Fluorinated Graphite and Bioactive Glass-Containing Orthodontic Bonding Resin

**DOI:** 10.3390/ma12081308

**Published:** 2019-04-22

**Authors:** Hyung-Jin Nam, You-Min Kim, Yong Hoon Kwon, In-Ryoung Kim, Bong-Soo Park, Woo-Sung Son, Seung-Min Lee, Yong-Il Kim

**Affiliations:** 1 Department of Orthodontics, Dental Research Institute, Pusan National University Dental Hospital, Yangsan 50612, Korea; onnuri00@gmail.com (H.-J.N.); yumini78@naver.com (Y.-M.K.); wsson@pusan.ac.kr (W.-S.S.); 2 Department of Dental Materials, School of Dentistry, Pusan National University, Yangsan 50612, Korea; y0k0916@pusan.ac.kr; 3 Department of Oral Anatomy, School of Dentistry, Pusan National University, Yangsan 50612, Korea; biowool@pusan.ac.kr (I.-R.K.); parkbs@pusan.ac.kr (B.-S.P.); 4 Institute of Translational Dental Sciences, Pusan National University, Busan 46241, Korea

**Keywords:** graphite fluoride bioactive glass, remineralization, bioactive glass, white spot lesion

## Abstract

All orthodontic appliances are potentially cariogenic. The plaque around the orthodontic appliance can make demineralization on tooth surface causing white spot lesion (WSL). The most effective method to prevent WSL is Fluoride appliance and gargling, but this requires patient cooperation, which consumes additional treatment time and cost. As suggested in this study, biomaterials like bioactive glass and fluorinated graphite (FGt) having antibacterial and anti-demineralization ability effective and easy to use in the clinic. To clinically use orthodontic bonding resins containing Graphite Fluoride BAG (FGtBAG), its properties, biological stability, antimicrobial activity, and remineralization effect must be verified. BAG was mixed with 2.5% FGt containing 51 to 61% fluorine. This mixture was mixed with the CharmFill Flow (CF) in the ratios of 1, 3, and 5 wt%. Microhardness and shear bond strength tests were performed to evaluate its mechanical properties. MTT (3-(4, 5-dimethyl thiazol-2-yl)-2, 5-diphenyl tetra) assay was performed for evaluating its safety. *Streptococcus mutans*, which is major cariogen by producing lactic acid, was evaluated for antibacterial ability of reducing WSL. In addition, x-ray images were obtained by CBCT (Cone beam computed tomography) after a pH cycle. The remineralization effect was verified in vivo and by Image J. FGtBAG did not differ significantly from CF in mechanical tests. The MTT assay found no significant differences between the groups. The antibacterial activity of FGtBAG at 24 h and 48 h was significantly higher than that of CF. The fluoride release rate tended to increase with the FGtBAG content. The pH cycle results showed that FGtBAG had higher concentration-dependent remineralization effect than CF. The results of this study suggests that orthodontic resins containing FGtBAG can prevent WSL owing to their antibacterial activity and remineralization effect.

## 1. Introduction

White spot lesions (WSLs) often damage the aesthetics outcome on orthodontic patients. The demineralization by WSL induce the chalky and white surface on the enamel surface [1]. The opaque surface of the WSL is clearly visible to the naked eye. In particular, WSLs often occur on the upper anterior teeth, where they affect the aesthetics and may progress to caries [2]. The mouth cavity contains microorganisms such as fungi, archaea, and viruses and bacteria [3]. While some microorganisms have a positive effect on the host, there are bacteria threaten health care such as caries, gingivitis and periodontitis [4]. Bacteria are a major cause of the negative effects associated with teeth. Bacteria that cause cavities on the tooth surface are as follows. The bacteria such as *non-mutans streptococci, Streptococcus sanguinis, Actinomyces* is on the sound surface. The *Streptococcus mutans* begin to appear on the mature plaque [5]. The main cause of the demineralization at the WSL is bacteria such as *S. mutans* and lactobacilli induced low pH. 

Bacteria such as *S.mutans* produce the lactic acid which is dissolute the tooth mineral (hydroxyapatite, Ca_10_(PO_4_)_6_(OH)_2_). Orthodontic appliances generate rough surfaces, where plaques accumulate. Poor oral hygiene and irregular dental surfaces during orthodontic treatment encourage the bacteria growth causing the enamel surface demineralization [6].

Patient training and periodic fluoride applications have been used to prevent WSLs. In general, the orthodontic patients were trained for tooth brushing. However, young patients do not follow this instruction. Thus, the method to prevent WSL without co-operation is absolutely necessary. Recently, studies were conducted on adding biomaterials in the bonding agent to prevent WSLs without the need for patient cooperation or additional chair time [7,8,9]. The biomaterials on bonding agents must be no harm to the human body and must not inhibit the physical properties of the bonding agent after addition. Furthermore, they should perform functions such as inhibiting the growth of bacteria that decrease the pH and result in demineralization, or ion releasing to raise the pH.

One biomaterial that has been researched intensively in recent years is bioactive glass (BAG). BAG has the Si-O-Si basic structure and is composed of Na_2_O, P_2_O_2_, and CaO. When BAG is added to a resin paste, it plays the role of a filler in addition to having a buffer effect owing to its antibacterial and ion releasing properties. BAG creates a super saturated ion state by releasing Na^+^, Ca_2_^+^, and PO_4_^3-^ ions in a liquid environment. The released ions change the precipitated amorphous calcium phosphate layer into apatite, increase the pH, which has been already decreased by oral bacteria, and show an antibacterial effect [10]. As the graphene-based materials have the osteogenic inducing ability of stem cells and biocompatibility, it is drawing attention as biomaterials. Graphite fluoride, also called polycarbon monofluoride, is a graphite-based compound having a fluorine-containing platelet structure. Graphite has recently been found to have antibacterial effects, particularly against dental pathogens. The antibacterial activities of graphene-based materials are widely known [11]. According to Sun et al., when adequate quantity of fluorinated graphene was added to glass ionomers, the physical properties improved and fluorine ions were released [12]. Fluorine re-mineralizes the teeth and exterminates bacteria, as it changes the essential enzyme activity by the penetration of HF (Hydrogen fluoride) into cells [13]. This study is expected to reduce WSL by fluoride releasing through routine bonding processing without extra cost and time with proper clinical properties. This study examines the clinical applicability of a biomaterial produced by mixing FGt and BAG as an orthodontic bonding resin with antibacterial activity and remineralization effect.

## 2. Materials and Methods

### 2.1. Synthesis of Graphite Fluoride BAG (FGtBAG)

BAG synthesis process is sol-gel method as follows [14]. First, 23 mmol of tetraethyl orthosilicate (Sigma-Aldrich, St. Louis, MO, USA) was mixed with 24 mL of ethanol. The pH of the solution was adjusted to 1–2 using 1N HNO_3_ (Samchun, Korea). Then, 14 mmol of Ca(OH)_2_ (Sigma-Aldrich, MO, USA) was added to synthesize BAG. NaOH (11.5 mmol) was added to the solution. A solution produced by melting 1.25 mmol of (NH_4_)_2_HPO_4_ in 400 mL distilled water (DW) was added. The solution pH was adjusted to 11 with ammonia solution. Then, DW was blended to obtain total volume of 600 mL. The solution was stirred for 48 h and then dried for 24 h. Finally, the sample was treated in a furnace at 600 °C for 6 h.

For FGtBAG, 2.5 wt.% of graphite fluoride (ACS, Pasadena, CA, USA) was added to the synthesized BAG and physically mixed twice for 10 s each time using a mixer (TORNADO SHM-ALM00, Shinhung, Korea). The F/C ratio of graphite fluoride was 0.8–1.1, and the F content was 56–61%. It was a homogeneous mixture. 

### 2.2. Characterization of FGtBAG

FGtBAG was observed using field-emission scanning electron microscopy (FESEM, MIRA3, TESCAN, Brno, Czechia).

The X-ray diffraction (XRD, Ultima 4, Rigaku, TX, USA) patterns of BAG and fGtBAG were analyzed with Cu Kα radiation (λ = 1.5409292Å) at 40 kV and 40 mA (The step size: 0.020°, the scanning rate: 1.50° s^−1^ in the 2θ range of 10 to 50°).

The typical functional groups of BAG and fGtBAG were analyzed in the range of 400–4000 cm^−1^ using the KRr method with Fourier transform infrared spectroscopy (FT-IR, Spectrum GX, PerkinElmer, Wellesley, MA, USA).

### 2.3. Preparation of the FGtBAG-Containing Orthodontic Bonding Rsion Disk

Resin disks (Φ: 5 mm, thickness 2 mm) were fabricated to estimate FGtBAG-containing orthodontic bonding resin. FGtBAG was added to 2 mL of the orthodontic bonding resin (CF, CharmFill Flow, Dentkist, Korea) in 2 mL black e-tubes to achieve the FGtBAG content of 1, 3, and 5 wt.%. Then, they were mixed twice for 10 s each time using a mixer (TORNADO SHM-ALM00, Shinhung, Seoul, Korea). The evenly mixed samples were injected into brass molds, which were covered with a slide glass (t: 0.2 mm) and photopolymerized for 20 s VALO (Ultradent Products, South Jordan, UT, USA) (Table 1). 

### 2.4. Microhardness

The prepared disk (orthodontic bonding resin) was tested with a microhardness testing machine (MVK-H1, Akashi, Japan) by applying a load of 1.96 N on top of the disk. Three specimens were used for each group and each sample was measured three times.

### 2.5. Shear Bond Strength (SBS)

Shear bond strength was measured with the Instron machine (Instron Corporation, Canton, MA, USA) to evaluate the bracket adhesion of the synthetic bonding resin. The premolars were used for each group (n = 5). This research was approved by the Institutional Review Board of Pusan National University Dental Hospital (PNUDH-2018046). Premolars with no WSL and no other enamel defects were used in this test. The tooth surface was washed with no-fluorine pumice washed with DW for 10 s, and dried. They were etched for 15 s using a 35% phosphoric acid, sucked, washed with water, and then dried. Orthodontic primer (Transbond™ XT adhesive primer, 3M, Nonrovia, CA, USA) was applied to the premolar surface and air was blown over it for 2 s gently. The brackets (Damon orthodontic metal standard edgewise brackets, Ormco, CA, USA) were bonded on tooth surface. The remaining paste was eliminated and then the mesial and distal sites were photopolymerized for 5 s. This entire process was conducted as per the recommendations of the CharmFill Flow manufacturer. The bracket bonded premolar tooth was kept in DW for 24 h and then analyzed using Instron (Crosshead speed: 1 mm/min). 

### 2.6. Antibacterial Test

The *S. mutans*, the major etiological agent of WSL on the bacterial field, was used for the antibacterial test. In order to investigate the antibacterial effect in the bonding agent, the resin disk as mentioned in 2.3 was tested as follows. The disks were placed in 96 well plates and bonded to the bottom plate by photopolymerization with the same resin as used for the control group. 96 well plates were used in this experiment after low-temperature plasma sterilization (LOWTEM Crystal 50, Gunpo-si, Korea). *S. mutans* was put in a brain heart infusion medium at the concentration of 1.0 × 10^5^ CFU/mL, and was cultured in an incubator at 37 °C. The absorbance was measured at 620 nm after culturing for 24 h and 48 h.

### 2.7. MTT Assay

MTT (3-(4,5-dimethyl thiazol-2-yl)-2,5-diphenyl tetrazolium bromide)-assay was performed to evaluate the cytotoxicity of the orthodontic bonding agents containing fGtBAG. The orthodontic brace was placed on the tooth surface excessive bonding agent often flows on the gingiva. So human gingival fibroblasts-1 (HGF-1; ATCC, Rockville, MD, USA), the most abundant cell in periodontal connective tissue, used for cell viability test. The resin sample disks were inserted into 96 well plates and sterilized with low-temperature plasma (LOWTEM Crystal 50, Gunpo-si, Korea). HGF-1were cultured in Dulbecco’s modified Eagle’s medium (Hylone, Logan, UT, USA) containing 10% fetal bovine serum (Hyclone, Logan, UT, USA) and 100 IU/mL penicillin/streptomycin (Hyclone, Logan, UT, USA). The HGF-1 cells were injected into 96 well plates containing the samples and cultured in a 5% CO_2_ incubator at 37 °C for 24 h. Then, MTT 3-(4,5-dimethylthiazol-2-yl)-2,5-diphenyltetrazolium bromide (Sigma-Aldrich, St. Louis, MO, USA) was added at a concentration of 5 mg/mL and reacted for 4 h in a dark room. The supernatant was removed and the samples were melted with MTT crystal dimethyl sulfoxide (DMSO; Sigma-Aldrich, St. Louis, MO, USA, 150 µl/well) formed in the cells. The absorbance was measured at 620 nm wavelength (Sunrise^TM^, TECAN, Männedorf, Switzerland).

### 2.8. In Vitro Fluorine Dissolution Test

The fluorine ion releasing test was performed by measuring the ion dissolution from the resin disk contained in the simulated body fluid (SBF, Biosesang, Seongnam-si, Korea). Ion chromatography (ICS-5000, ThermoFisher-Dionex, Sunnyvale, MA, USA) was performed to evaluate the F ion release capacity of the sterilized resin disk. The sterilized resin disk and 5 mL of SBF were stored in a 5-mL tube for 0.5, 5, 10, and 20 days [15]. The concentrations of ions released in the resin disk were measured.

### 2.9. Remineralization Test

The pH cycling protocol was proposed to evaluate the remineralization capacity of the orthodontic bonding agents containing FGtBAG [16]. Teeth extracted for orthodontics were used in this experiment. Five premolars with no WSL or other enamel defects were used for each group. This study was approved by the Institutional Review Board of Pusan National University Dental Hospital (PNUDH-2018046).

**Figure 1:**
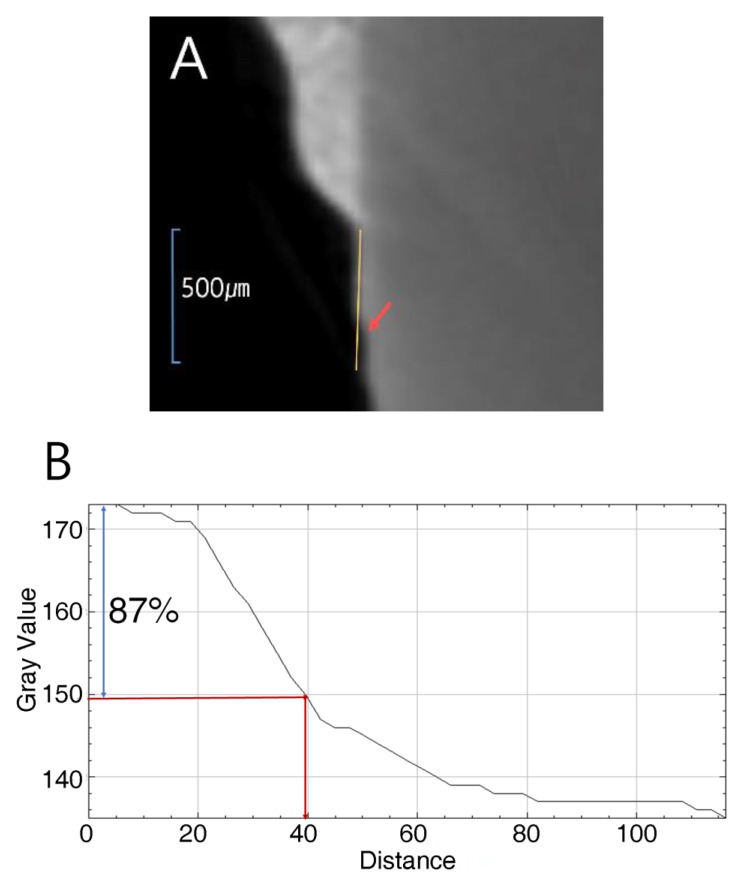
Remineralization length measurment. (**a**) CBCT slice of the region of interest on the enamel surface; the end of the bonding resin was starting point; yellow line: region of interest to the reference point on the enamel surface; (**b**) histogram. Blue arrow: up to 87% level of gray value from the reference point; red arrow: the distance to the 87% gray value from the reference point.

The pH cycle for remineralization evaluation was as follows. Tooth samples for test was buried in acrylic resin mold. The tooth surfaces of the buried tooth samples that were to be bonded were washed with no-fluorine pumice washed with water for 10 s, and then dried. A nail varnish was clearly marked to the top of the 5 mm × 5 mm rectangular vertex to prevent etching except for the 5 mm × 5 mm tooth surface. The tooth surface was etched for 30 s with 35% phosphoric acid washed with water for 10 s and dried. CF and CF with FGtBAG samples were applied to the tooth surface and photopolymerized for 5 s. After storing the teeth in DW for 24 h, they were settled alternately in a demineralization solution (Biosesang, Seongnam-si, Korea) and a remineralization solution (Biosesang, Seongnam-si, Korea) each 6 and 18 h. This repeated cycle continued for 14 days. The solutions changed with fresh solutions every week. In between the transfer from the demineralizing solution to the remineralizing solution, the samples were washed with DW for 1 min and dried before changing the solution every day. The samples were measured using µ-CT (90 KV and 109 µA, InspeXio, Shimadzu, Kyoto, Japan). The measured µ-CT data were analyzed using ImageJ (National Institutes of Health, Bethesda, Md) [17] (Figure 1). The lengths in Image were corrected with a scale bar on the µ-CT. Using the brightness on histogram, sound enamels were defined by brightness of up to 87%. The distance from the point where the difference was larger than 87% of the sample was measured and defined as the remineralization length.

### 2.10. Statistical Analysis

One-way analysis of variance (ANOVA) used to analyze the differences among group means in the sample. A class of post hoc tests was Duncan’s Test; examined properties include microhardness, shear bond strength, antibacterial test, cell viability test, and pH cycle test. ARI was verified with the Kruskal-Wallis test. Every statistical analysis was performed with R language program (version 3.6.0; R Foundation for Statistical Computing, Vienna, Austria).

## 3. Results

### 3.1. Characterization

**Figure 2:**
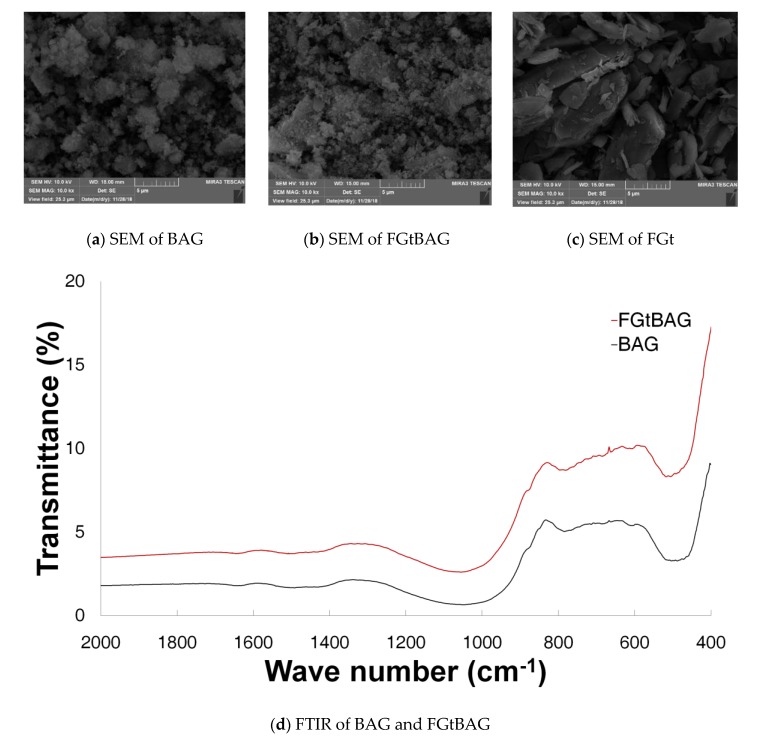

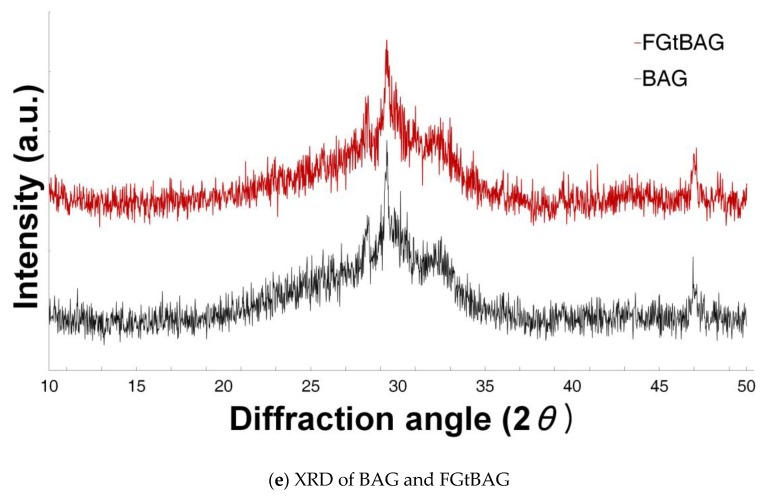
BAG and FGtBAG characterization. SEM images of (**a**) BAG; (**b**) FGtBAG; (**c**) FGt; (**d**) FTIR of BAG and FGtBAG; and (**e**) XRD of BAG and FGtBAG.

Generally, polygonal particles seen in the bioactive glass were observed in the SEM of the synthesized BAG and FGtBAG. The same plate structure as shown in the SEM of the FGt could be seen in the image of the FGtBAG. In this study, aggregation of particles could be seen in the images of BAG and FGtBAG [18] (Figure 2). In the FTIR, Si-O-Si bond vibration could be observed at 540–470 cm^−1^ [18]. No crystalline peak was observed in the XRD pattern [7].

### 3.2. Microhardness

**Figure 3:**
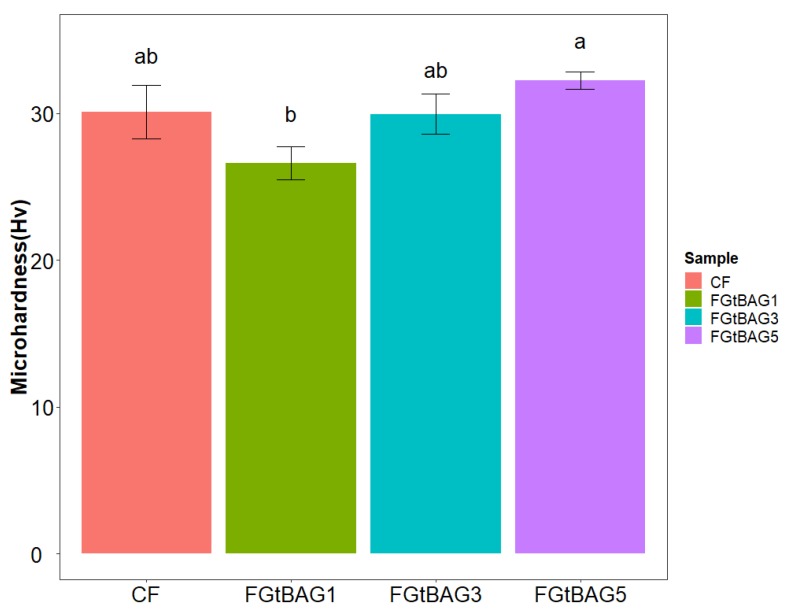
Microhardness comparison of CF and FGtBAG samples. The different letters indicate significant difference between the groups (*p* < 0.05) by Duncan’s multiple comparison test. Error bars are shown as ± standard errors (n = 5).

The CF group (30.1 ± 1.8 Hv) showed no significant differences between FGtBAG1 (26.6 ± 1.1 Hv), FGtBAG3 (30.0 ± 1.4 Hv), and FGtBAG5 (32.2 ± 0.6 Hv) (Figure 3).

### 3.3. Shear Bond Strength (SBS)

**Figure 4:**
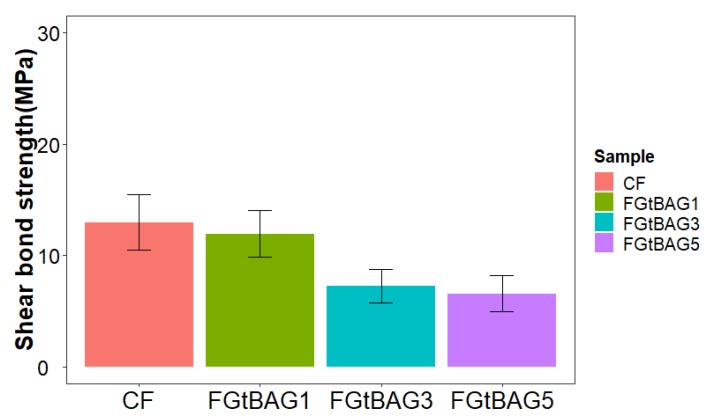
SBS comparison CF and FGtBAG (*p* > 0.05) by Duncan’s multiple comparison test. Error bars are shown as ± standard errors (n = 5).

The CF (13.0 ± 2.5 MPa) showed no significant differences between FGtBAG1 (12.0 ± 2.1 MPa), FGtBAG3 (7.2 ± 1.5MPa), and FGtBAG5 (6.6 ± 1.6 MPa) (*p* > 0.05) (Figure 4).

### 3.4. Adhesive Remnant Index (ARI) Score

There was no significant difference in the ARI scores among CF (3.6 ± 0.9), FGtBAG1 (4.0 ± 0.0), FGtBAG3 (3.6 ± 0.9), and FGtBAG5 (3.6 ± 0.5) (Table 2). This result was shown that control CF and FGtBAG containing resin group was not different on tooth surface adhesion. 

### 3.5. MTT Assay

**Figure 5:**
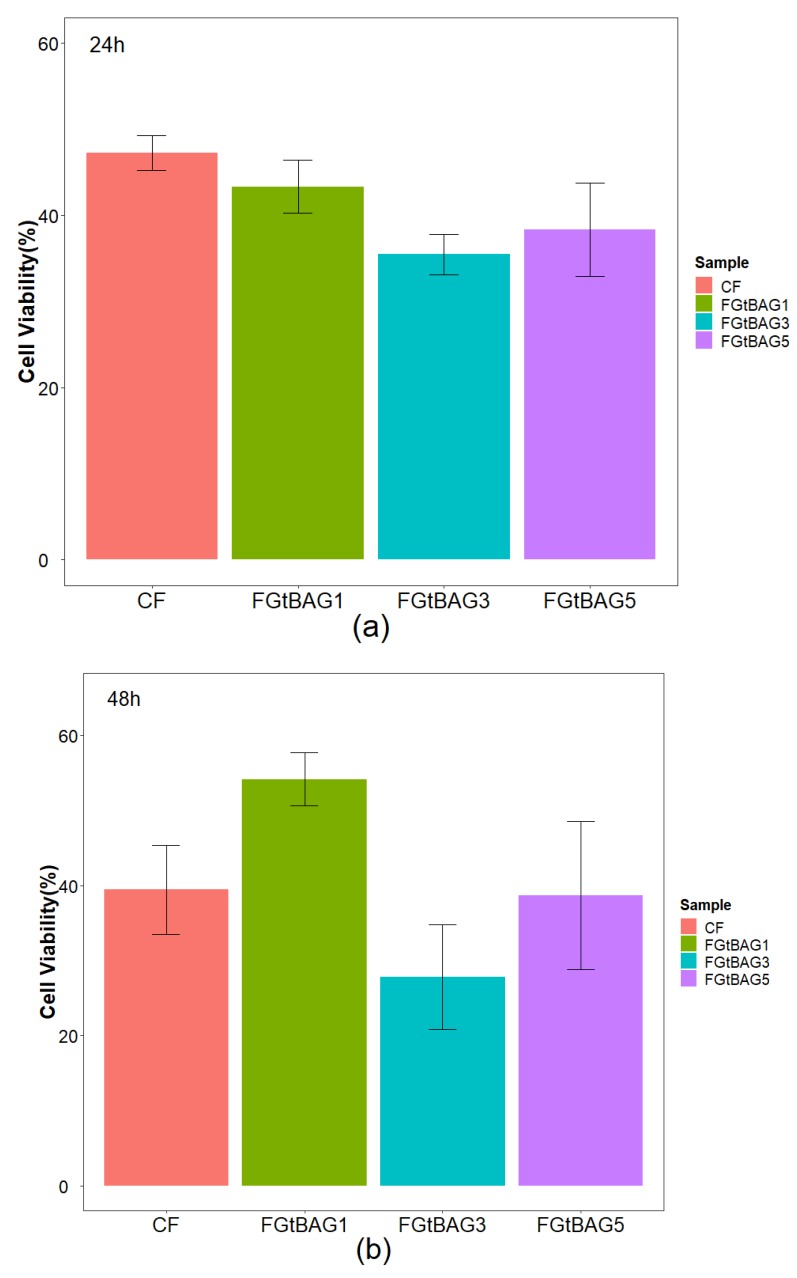
Results of MTT assay on cured CF and FGtBAG—containing orthodontic bonding resin after (**a**) 24 and (**b**) 48 h. The different letter indicates statistically significant difference between the groups (*p* > 0.05) by Duncan’s multiple comparison test. Error bars are shown as ± standard errors.

The MTT assay results after 24 h (Figure 5a) showed no statistically significant differences between CF (42.3 ± 2.0%), FGtBAG1 (43.3 ± 3.1%), FGtBAG3 (35.5 ± 2.3%), and FGtBAG5 (38.3 ± 5.4%). Similarly, the MTT assay results after 48 h (Figure 5b) showed no statistically significant differences between CF (39.4 ± 5.9%), FGtBAG1 (54.1 ± 3.5%), FGtBAG3 (27.8 ± 6.9%), and FGtBAG5 (38.7 ± 9.9%). These results show that there is no difference in the cell viability test between the commercially available product and the orthodontic bonding agent used in this experiment.

### 3.6. Antibacterial Test

**Figure 6:**
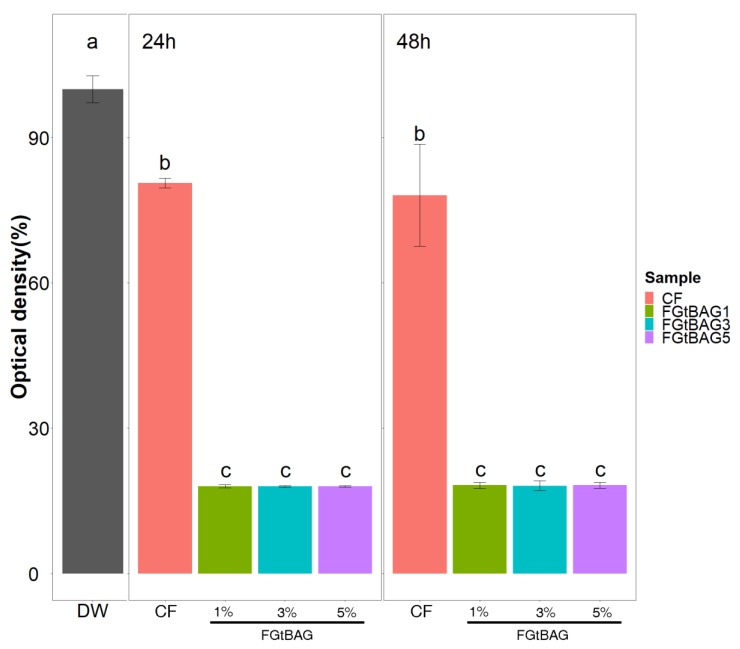
Difference between antibacterial properties of cured CF and FGtBAG at 24 and 48 h. The different letter indicates statistically significant difference between the groups (*p* < 0.05) by Duncan’s multiple comparison test. Error bars are shown as ± standard errors.

Statistically, FGtBAG1 (18.0 ± 0.4%), FGtBAG3 (18.0 ± 0.1%), and FGtBAG5 (18.0 ± 0.1%) groups showed significantly higher antibacterial capacity after 24 h with 100% DW when compared with the CF group (80.6 ± 1.0%; *p* < 0.001), as shown in Figure 6. The FGtBAG1 (18.3 ± 0.6%), FGtBAG3 (18.2 ± 1.0%), and FGtBAG5 (18.3 ± 0.6%) groups also showed statistically significantly higher antibacterial capacity after 48 h when compared with the CF group (78.1 ± 10.0%; *p* < 0.001).

### 3.7. In Vitro F Dissolution Test

**Figure 7:**
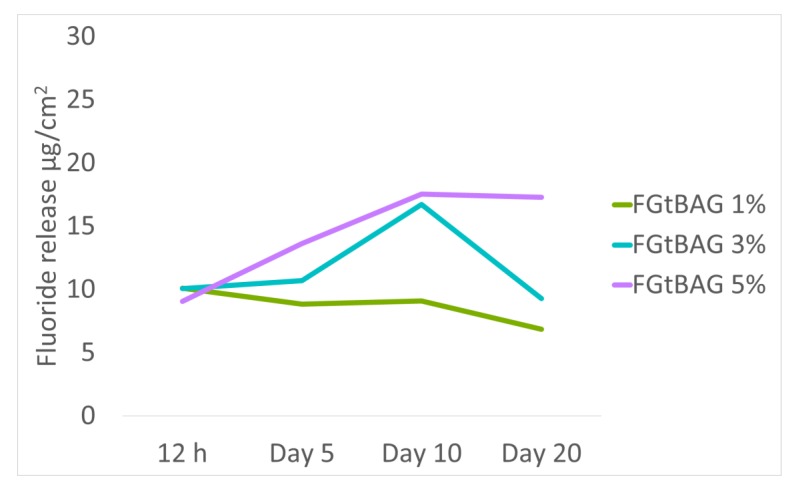
Fluoride release from 1, 3, and 5% FGtBAG. Resin disks were used to prepare discs of 5 mm diameter and 2 mm thickness.

FGtBAG1 showed fluorine ion release of 6.9–10.1 µg/cm^2^ from 0.5 day to 20 days. FGtBAG3 and FGtBAG5 showed fluorine ion release of 9.2–16.7 µg/cm^2^ and 9.1–17.3 µg/cm^2^, respectively. As the FGt content increased, the fluorine ion release rate also increased (Figure 7).

### 3.8. Remineralization

**Figure 8:**
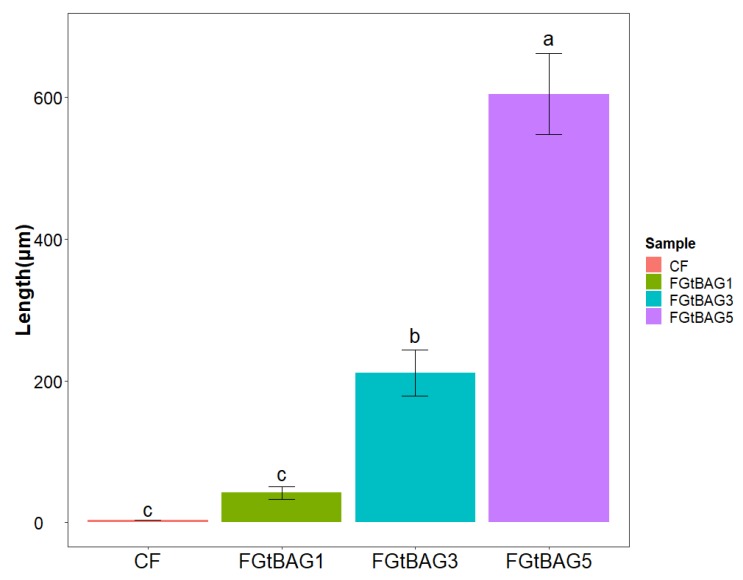
Remineralization length comparison of orthodontic bonding resin containing CF and FGtBAG by ImageJ analysis. The different letter indicates statistically significant difference between the groups (*p* < 0.05) by Duncan’s multiple comparison test. Error bars are shown as ± standard errors.

**Figure 9:**
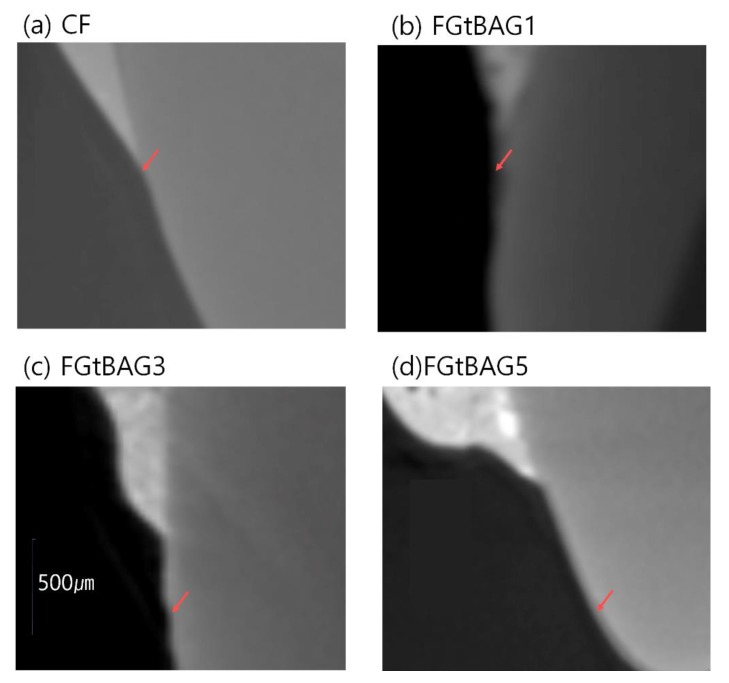
Remineralization points of the CF and FBAG orthodontic bonding resin via MicroCT. (**a**) CF; (**b**) FGtBAG1; (**c**) FGtBAG3; and (**d**) FGtBAG5.

The remineralization abilities of FGtBAG1 (41.9 ± 8.6 µg), FGtBAG3 (211.2 ± 32.3 µg), and FGtBAG5 (605.2 ± 126.8 µg) were significantly higher than that of CF (3.8 ± 0.0 µg), as shown in Figure 8. The remineralizarion ability increased remarkably with FGtBAG content. The remineralization points for each resin sample can be seen in Figure 9.

## 4. Discussion

WSL is a side effect that negatively affects the aesthetics of patients undergoing orthodontic treatment. Demineralization occurs on the enamel surface of teeth due to excessive etching during the process of attaching a fixture, proliferation of bacteria, and a decrease in pH because of the formation of plaques around the orthodontic devices. The opaque lesions caused by surface demineralization should be prevented because they not only degrade the aesthetics, but they can also progress to caries. To prevent WSLs, patients are given instructions on tooth brushing (TBI) after attachment of orthodontics, but this requires patient cooperation. Moreover, it is difficult to prevent WSLs in young patients because their level of cooperation is low. The antibacterial and remineralization effects of gargling with fluorine have been demonstrated, but this method has disadvantages because it requires patient cooperation along with additional treatment time and cost [6,13].

In contrast, the addition of biomaterials to orthodontic bonding materials, which is a new area of research, yields physically stable and biologically safe technical solutions and results. Such materials have shown the ability to decrease WSLs owing to their antibacterial activity and remineralization effect [7,8]. In this study, the experimental samples that had FGtBAG added to the orthodontic bonding material instead of CF showed increased concentration-dependent microhardness, but this difference was not significant.

When compared with CF, the SBS of FGtBAG was slightly decreased, but the difference was not significant. Although statistically insignificant, a lower SBS could be due to darker and low polymerization [8]. Therefore, polymerization for a longer time will be necessary in actual clinical settings.

In the biological evaluation, the MTT assay results of the experimental materials did not show statistically significant differences from those of CF at both 24 h and 48 h. Graphite is a safe biomaterial that has been researched for a long time with regard to osteoinductive factors in the tissue engineering field [19,20]. F has toxicity, but the F in FGt used in this study is believed to have low toxicity as its amount of release in 20 days was only 6.9–17.3 µg/cm^2^.

When the antibacterial activity was examined, the experimental materials showed much higher antibacterial activity than CF at both 24 h and 48 h. BAG has antibacterial activity because of the exchange function of the released ions (e.g., Na^+^, K^+^, Ca^2+^) with H^+^ and the increase in osmotic pressure. Furthermore, the released F^−^ penetrates through the cell walls as HF. The penetrated HF is changed to H^+^ and F^−^ and lowers the PH in the cells. The low pH changes the essential enzyme activity in the cells, which kill the bacteria [6,13]. Antibacterial activity of graphene-based materials are well known [11]. 

When the remineralization effect of the experimental materials was examined through the pH cycle, a concentration-dependent remineralization effect was shown. This is believed to be because of the buffering effect caused by the ion release in BAG [21] and the HAP (hydroxyapatite) formation. As the concentration of FGtBAG increases in orthodontic bonding resin, ion (Ca2+, and PO_4_^3−^) released from BAG increased. The increased ion formed high concentration ion lay around the bracket. The increased ion prevented the demineralization of hydroxyapatite in an acidic environment during the pH cycling [10]. Al-Eesa et al. reported that when FBAG was used, HAP was formed after 24 h and the HAP formation was promoted by the F ions [22]. In summary, FBAG releases more ions in an acidic environment and forms HAP. Thus, it has the advantage of releasing more ions when a clinical acidic environment is formed.

## 5. Conclusions

FGtBAG in orthodontic bonding resins has physical stability and biological safety necessary for clinical use. FGtBAG shows higher concentration-dependent antibacterial activity than CF. FGtBAG has greater remineralization effect than CF. The orthodontic bonding resins containing FGtBAG showed the potential for preventing WSLs. This study result showed that the orthodontic bonding resin containing FGtBAG have a potential of clinical usage to prevent WSL. This study result showed that the orthodontic bonding resin containing FGtBAG have a potential of clinical usage to prevent WSL.

## Figures and Tables

**Table 1:** Fluorine content in CF and FGtBAG.

Sample	CF	FGtBAG1	FGtBAG3	FGtBAG5
Fluorine contents in resin	0	1.4–1.5 ppm	4.2–4.5 ppm	7.0–7.5 ppm

**Table 2:** Adhesive Remnant Index (ARI) scores *^,^ **.

Sample	CF	FGtBAG			Significant
		1%	3%	5%	
Mean (SD)	3.6 (0.9)	4.0 (0.0)	3.6 (0.9)	3.6 (0.5)	Not significant
Median, Q1–Q3	4 (3-4)	4 (4-4)	4 (3-4)	4 (3-4)	
Min.–max.	2-4	4-4	2-4	3-4	

* ARI scores were analyzed by the Kruskal–Wallis test at α = 0.05 (n = 5). ** Score 5: No adhesive remained on the tooth, Score 4: Less than 10% of the adhesive remained on the tooth; Score 3: Between 10–90% of the adhesive remained on the tooth; Score 2: More than 90% of the adhesive remained on the tooth; Score 1: The entire adhesive remained on the tooth.
